# The experience of dysmenorrhoea among Ghanaian senior high and university students: pain characteristics and effects

**DOI:** 10.1186/1742-4755-11-58

**Published:** 2014-07-26

**Authors:** Lydia Aziato, Florence Dedey, Joe Nat A Clegg-Lamptey

**Affiliations:** 1 Department of Adult Health, School of Nursing, University of Ghana, P.O. Box LG 43, Legon, Accra, Ghana; 2 Department of Surgery, University of Ghana Medical School, Accra, Ghana

**Keywords:** Qualitative research, Phenomenology, Ghana, Menstrual pain, Adolescents

## Abstract

**Background:**

Dysmenorrhoea is a common problem of women at the reproductive age and may have negative effect on the education of females at various stages on the educational ladder.

**Context and purpose:**

This study sought to gain an in-depth understanding of the experience of dysmenorrhoea and its effect on female students in a secondary and a tertiary institution in Accra, Ghana.

**Methods:**

The study employed a descriptive phenomenology design and was conducted at a University and a Senior High School (SHS) in Accra. Purposive and snowball sampling techniques were used to recruit participants and data was saturated with 16 participants. Concurrent analysis was done by applying the processes of content analysis and the NVivo software was used to manage the data.

**Results:**

It was realized that dysmenorrhoea is associated with symptoms such as diarrhoea, headache and vomiting. Pain may start one week to the day of menstruation and the severity differed across the days of menstruation. The effect of dysmenorrhoea included activity intolerance, altered emotion and interaction, altered sleep pattern, absenteeism and inattentiveness, wishes and regrets, and misconceptions.

**Conclusions:**

It was concluded that severe dysmenorrhoea has a debilitating effect on female students and is associated with misconceptions that could result in drastic action with fatal consequences. Thus, there is the need to enhance education on dysmenorrhoea, and an aggressive step should be taken to effectively manage dysmenorrhoea.

## Introduction

Dysmenorrhoea refers to painful menstruation and it is common among adolescents and young women. The incidence reported ranges between 40 to 80% in different countries including Malaysia, Nigeria, and Ghana [1-6]. Dysmenorrhoea may start a few days before menstruation and lasts for a few hours to several days. Primary dysmenorrhoea may commence with menarche or within a year of menarche whilst secondary dysmenorrhoea begins several years after menarche. Primary dysmenorrhoea is common among adolescents and has no pathological associations. Primary dysmenorrhoea has been associated with increased prostaglandins [7]. However, secondary dysmenorrhoea has underlying disease [5]. Some women are relieved of the problem after having their first child and others experience the problem until their menopause. Dysmenorrhoea may be classified into mild, moderate and severe, depending on the degree of pain experienced and the debilitating effects of the problem [2,8].

Dysmenorrhoea may be associated with headache, diarrhoea, nausea and vomiting and these problems may occur prior to menstruation or with menstruation [5]. The pain may radiate to the thigh or the lower back. Dysmenorrhoea may be described as crampy, shooting, or biting. The quality of pain varies among individuals and those with severe pain have more negative effects [5,9,10]. It is emphasised that pain is an individual phenomenon and defined as ‘whatever the experiencing person says it is and existing whenever the person says it does’ [11]. Therefore, the experience of dysmenorrhoea, although a global issue, has an individual connotation due to the personal nature of pain.

Severe dysmenorrhoea has physical, psychological, and social consequences [5]. Pain is debilitating and impairs activities of daily living. The individual in pain becomes depressed and moody and this interferes with social interaction [2]. During this period, some adolescents and women lose their appetite and they may absent themselves from school or work [1-3]. In the United States of America, it is estimated that the state loses about two billion dollars a year due to the inability of women in pain to go to work [5]. The huge economic loss associated with dysmenorrhoea [12] makes it imperative for the phenomenon to be fully explored so that appropriate interventions can be implemented to curb the problem.

Few qualitative studies have explored in depth, the experiences of female students with dysmenorrhoea to gain full understanding of the phenomenon of dysmenorrhoea and the impact on the lives of this category of sufferers [13]. An in-depth qualitative exploration is required for a personal phenomenon such as dysmenorrhoea so that appropriate interventions can be instituted. Little is known about pain description and the effect of dysmenorrhoea among Ghanaian female students within the educational institutions. This article focuses on pain description and its effect and it is drawn from a wider study aimed at exploring issues relating to dysmenorrhoea within the educational system in Ghana.

## Methods

### Design and setting

The study adopted a descriptive phenomenology to enable a full understanding of the experiences of female students with dysmenorrhoea. Phenomenology is appropriate to examine the lived experiences of a phenomenon [13].

The study was conducted in a University and a girls’ Senior High School (SHS) in Accra, Ghana. The University admits both male and female students and runs several programmes ranging from certificate to doctoral programmes across disciplines. The SHS involved in this study admits about a thousand students and runs courses in the sciences, business, arts, and home economics. Both institutions in this study are state-owned.

### Sampling and data collection

The study targeted female students with dysmenorrhoea. The inclusion criteria were female students who experience dysmenorrhea and were students of the two schools. Purposive and snowball sampling techniques were employed and data saturation was achieved with 16 participants (8 SHS students and 8 University students).

Data collection was done through in-depth individual interviews using a semi-structured interview guide. The interview explored the onset and duration of dysmenorrhoea, description of pain and the effects of pain. Follow-up questions were asked to gain full understanding of participants’ experiences. All the interviews were conducted in English, audio-taped with a digital audio recorder and transcribed verbatim. Interviews were conducted at a place and time convenient to the participants. Interviews lasted for 45 minutes to 60 minutes and were conducted by the first author who is experienced in qualitative interviewing. Participants consented to the recording of interviews.

### Ethical considerations

Ethical approval for the study was obtained from the Noguchi Memorial Institute for Medical Research for a larger study aimed at validating a context appropriate pain assessment scale for clinical use in Ghana. Permission was sought from the relevant gate-keepers at the two schools and all participants gave informed consent before interviews were conducted. Anonymity and confidentiality was assured and observed. Participants were assigned identification codes and these were used to present findings. All identifying information in participants’ accounts was removed to ensure anonymity. Interviews were stopped when participants became emotional during the interview and continued when they later consented to do so. The service of a counsellor was made available to participants at no cost to them but none of the participants used this service.

### Rigour

The processes undertaken to ensure rigour in this study included member checking where participants were asked to confirm interpretation or understanding gained from their accounts. Prolonged engagement was employed to ensure that detailed accounts were obtained in this study. Also, detailed audit trail was maintained to enable other researchers to verify processes undertaken in this study.

### Data analysis

Data were analysed concurrently employing the techniques of content analysis. Transcripts were read several times to immerse in participants’ world. Codes were identified and themes and sub-themes were generated. After this process, data was transported into NVivo software version 9 and this was used to manage the data subsequently. The researchers discussed the themes and sub-themes and where discrepancies occurred, a consensus was reached to ensure that participants’ experiences were captured faithfully. Verbatim quotes were used to support findings.

## Results

### Demographic characteristics

The participants were made up of 8 students at SHS and 8 students at the University. The SHS participants were aged between 16 and 18 years and were in the first and second years. Seven of the University students were in the final year of their undergraduate programme and were aged 22 to 30 years. One was a doctoral student aged 38 years. Two of the University students were married and one had two children. All the other participants were not married and they were all in school at the time of study. Fifteen participants were Christians and one was a Muslim.

The study revealed themes and sub-themes on the lived experiences of dysmenorrhoea as: pain characteristics (pain onset and duration, pain description, associated signs and symptoms), pain effects (activity intolerance, altered emotion and interaction, altered sleep pattern, absenteeism and inattentiveness, wishes and regrets, and misconceptions).

### Pain characteristics

This theme describes the characteristics of dysmenorrhoea experienced by participants. It was realized that the onset and duration of pain varied among participants, and some experienced these as warning signs of menstruation. Menstrual pain was associated with other symptoms such as vomiting, headache and diarrhoea. The sub-themes described are: the onset and duration of dysmenorrhoea, the description of pain, and other associated signs and symptoms.

### Pain onset and duration

Participants reported varied onset of dysmenorrhoea. Some said their pain starts about a week before the onset of menstruation – *“the pain starts about a week to my menses”* (UD4). Others said their pain starts on the day they start menstruation – *“the day the menses starts, I feel a lot of pain”.* (UD3) Also, a participant reported that her pain starts about an hour after the onset of menses – “*when I see the blood it takes about an hour before the pain starts”.* (UD15) The pain experienced before the onset of the menstrual flow affected different parts of the body such as the head, lower abdomen or the back:

*“…2 days before I have my menses I get a headache”* (SD10); *“…about a week to my menses, I have back pain which starts gradually; …and getting to the day of the menses, the pain is very severe”* (UD4);* three or four days before the menstruation I start experiencing lower abdominal pains”.* (UD5)

Some participants reported that pain started with their menarche or first menstruation – “*my menstrual pain started with my menarche”* (UD5) and others experienced pain after about a year after menarche – “*the pain started when I was about 13 years; a year after I started menstruating”.* (SD14) However a participant at the University reported that her pain subsided when she was 24 years old –“*I have been experiencing severe dysmenorrhoea since I started menstruating as a teenager at age 12; but the pain subsided miraculously at the age of 24”. (UD16)*

The duration of pain of the participants was variable, between a few hours to 5 days:

UD16: “*my pain lasts for 5 days”.*

UD1: *“… the first 3 days of my period is hell”*. (UD1)

SD13: *“… the severe pain takes two days but subsides on the third day”.*

SD10: *“…the first day is really painful but on the second day it gets better”.*

UD15: “*the funny thing about my pain is that, when it starts in the morning, roughly by 6 hours it just vanishes”.*

SD14: “*the pain is most severe the second and third day; the first day is not that severe”*.

### Pain description

Participants described the intensity of pain and the nature or quality of pain. Pain was described as *“severe”; “very severe”; “unbearable”; “real pain”.* On a scale of 0–10, pain was rated 6 – 10 plus.

*“This is real pain, real pain! It’s like two fresh sores being sawed and you are telling me to keep quiet … maybe 10 plus”* (UD1); *“I will rate it around 7 or 8”* (SD12); *“it is the most severe pain I have ever felt in my life so I will rate it at 10”* (SD13); *“…the pain is very severe”.* (UD4)

The quality of dysmenorrhoea was described as *“cutting”; “radiating” “stabbing”; “pricking”; “pulling”; “cramping”; “burning”; and “inconsistent pain”*.

*“I feel like someone is stabbing me with a knife or like using a knife to cut me. … it feels like something is radiating in my abdomen from one side to the other”* (UD2); *“it feels like somebody is pulling me”* (SD10); *“…It’s like someone has put the hand inside my under* (vagina) *and the person is pulling something”* (UD3); *“the feeling is not consistent, it is on and off”.* (SD11)

Some could not have the appropriate word or words to describe the pain felt which further highlights the individual nature of the phenomenon of pain - *“I wish you were in me. I do not know the word to use to describe it”* (SD8); *I do not really know how to explain it”.* (SD11)

### Associated signs and symptoms

Dysmenorrhoea was associated with a number of problems such as “*nausea and vomiting”, “diarrhoea”, “headache”, “backache”, “joint pains”, “weakness”, “leg and thigh pain”, “dizziness”, “fainting attacks”, “excessive sweating”,* and *“loss of appetite”*. The severity of these associated signs and symptoms varied in duration and some participants experienced them only on the first day while others experienced it during the entire duration of menstruation.

*“I will be feeling the pain, my head will be aching and I will be running* (diarrhoea)*”* (SD10); *“I become very weak … I get waist pains … I lose appetite and cannot eat”* (SD11); *“…I get ill, I feel cold and dizzy”* (SD12); *“I feel so dull, tired and dizzy”* (SD13); *“I feel pain at my waist, joints and legs. Sometimes my palms sweat and become reddish”* (UD3); *“I vomit on the first day twice or thrice but on the subsequent days I do not vomit*” (UD16); *“…whenever I am feeling pain, I sometimes pass out”.* (SD9)

Some participants had dysuria with the dysmenorrhoea and hence they felt reluctant to pass urine.

*“I keep my urine for 3 to 4 hours …Sometimes I cry a lot during urination because the pain is so severe”* (UD1); *“…if I urinate, the pain becomes more severe and it has a burning sensation; … and just after the last drops of urine, I feel a very sharp pain, which subsides but the dysmenorrhea continues”.* (UD16)

Some also experienced increased heart rate and panic attacks when they were about to menstruate. Others felt depressed when their menses was due. Fear led to a change in a participants’ wedding date.

*“…because of the pain, when it gets to a day before my menses, I become scared suddenly and my heart beats faster as if something has happened. But nothing has happened except my menses coming the next day”* (SD8); *“when the time for my menstruation approaches I get depressed”.* (UD16)

Another participants said:

*“… it comes with some fear which disrupts my activities so I have to cancel all my schedules. I even changed my wedding date because of my menstrual pain; I could not use the date I really wanted for my wedding”.* (UD15)

### Pain effects

This theme describes the effect of dysmenorrhoea on participants physically, and psychosocially, and on their education and work. Dysmenorrhoea also affected the thought processes, beliefs, and perceptions of individuals and led to suicidal ideations. Some participants could not undertake their normal activities during the pain, they became irritable and were not able to relate well with friends and family. Dysmenorrhoea also led to alterations in the sleep pattern, and some participants were not able to go to class or lectures during the pain. Some regretted being females and believed if they had a baby, the dysmenorrhoea would stop. Others wished they were dead when the pain was unbearable. The sub-themes that emerged were activity intolerance, altered emotion and interaction, altered sleep pattern, absenteeism and inattentiveness, wishes and regrets and misconceptions.

### Activity intolerance

Dysmenorrhoea hindered activity level. Some participants could not sit, stand, or walk and had to be supported to the bathroom and assisted to bath. The pain also led to inability to cook and wash and perform other daily activities. The degree of activity intolerance varied.

*“I cannot sit; I will just be rolling and lying down”* (SD10); *“sometimes I feel this numbing pain in my lower abdomen that I find it difficult to even move my legs”* (SD8); *“I cannot walk, cook, wash or do anything; I will be squatting. Sometimes I try to walk to the bathroom …but I still needed somebody to assist me take my bath or else I sit on the pavement in the bathroom to bath”.* (UD16)

Participants with severe dysmenorrhoea were not able to assume one position during pain and changed positions with the hope that they will be comfortable in a particular position -*”…I cannot lie in one position so I turn from side to side thinking that a particular position will reduce the pain”.* (UD15)

Activity intolerance also varied across the days of menstruation - *“On the first day I am carried to the sick bay because I cannot walk; the next day, I manage to walk with support”.* (SD9)

### Altered emotion and interaction

Dysmenorrhoea affected the mood of participants and they were not able to socialize as they used to. They became *“irritable”, “quiet”, “sad”, “angry”,* and *“depressed”* when in pain. Some preferred to be alone and when they talked, they felt more pain and became stressed.

*“I do not want to talk. I will be very quiet; I cannot talk because when I talk, it hurts”* (SD14); *“I feel it is stressful talking and I feel lazy to talk”.* (UD16)

*“…my attitude changes; I get angry easily and I always want to be left alone”* (SD9); *“I tend to push people away; I become moody”* (UD15); *“I get irritated when someone comes around me, I get bored and ask the person to leave”.* (SD10)

Some participants did not answer their phones or watch television (TV) and preferred darker environment when they were in pain due to their altered emotions - *“…I turn my phone off and lock the door; I do not even want to see light; I switch off the lights and the television and stay in the dark”.* (UD15) However, a few of the participants felt watching TV or a movie with others could distract their attention from the pain and lift their mood - *“but sometimes I wish maybe my sister, mum, boyfriend, husband or whoever is around to at least sit down, talk and watch a movie or TV with me”.* (UD3)

Participants were not able to attend social gatherings and some could not go out with their partners when they were in pain - *“I could not attend my sister’s outdooring”* (UD15); *“I really wanted to go to a party with my boyfriend but I had to disappoint him because I was in pain and could not go”.* (UD3)

### Altered sleep pattern

Some participants slept for longer periods and others were unable to sleep during dysmenorrhoea. Also, some fell asleep when crying in pain and they did not want to be disturbed when they slept.

*“I sleep intermittently”* (UD6); *“I have sleepless night; I toss and turn and when I wake up my eyes are red”* (SD8); *I sleep a lot, sometimes the sleep makes me forget about the pain”.* (SD9)

The participants found it difficult to assume their normal positions during sleep and some slept on the bare floor - *“…when I lie on the floor, I feel better; …I hold my stomach and I use 2 or 3 pillows, I place my leg on one pillow and my stomach on another”.* (SD10)

### Absenteeism and inattentiveness

Dysmenorrhoea resulted in absenteeism from school. Some participants went to school but were unable to concentrate, while others slept in class.

*“When it happens, I miss class, I am always at the sickbay or the hospital;* (SD8); *“I sleep in class” (SD9); “I hardly focus in class when in pain. I cannot take my mind off the pain”.* (UD3)

Four of the University participants who were employed prior to pursuing further studies reported that although they tried to work with pain, they sometimes sought permission and stayed off work - *“…I try to work but most of the time a colleague helps me with my work while I rest or I ask permission and stay off”* (UD16); *“I cannot work actively”.* (UD3)

### Wishes and regrets

Most of the participants with severe dysmenorrhoea regretted that they were females and wished they were males - *“Sometimes I regret being a lady!”* (SD9); *“Sometimes I wish I were a boy”* (UD5); *“Sometimes I just wish I was not a girl”.* (SD8) Some participants with severe dysmenorrhoea indicated that they wished to get pregnant so that they will be free from pain. Some were told that when they have sex or a baby, the dysmenorrhoea will stop.

*“I spoke to one doctor and he said when one starts having sex and gives birth, the pain will reduce or stop; so I want to give birth”* (UD4); *“I wanted to have a baby whether I had gotten married or not. I did not care; I just wanted to get pregnant so that for 9 months the pain will stop”.* (UD16)

In some cases of severe pain, some participants even had suicidal thoughts or the desire to do something drastic to end their suffering. Some wished they “*were not even born”* when they were in such severe pain.

*“When I was in severe pain, I wish I was dead. I just wanted to sleep and not wake up again; … I really wanted to die”* (UD16); *“I wish I could use something to prick my stomach because it was so painful”* (SD7); *“Sometimes I just wish I was not born. I wanted to cut my abdomen to see the blood ooze out, so that I will be free”.* (SD8)

Others wished for an early menopause: “*I cannot wait to have my menopause so that it will go away since some of my cousins have pain after they had children”.* (UD6)

### Misconceptions

Some participants believed that dysmenorrhoea could render them childless: *“…sometimes I get scared because I heard you cannot give birth if you have dysmenorrhoea”* (UD15).

A participant reported that she was told a crab was at the lower abdomen causing the pain. - *“When I was younger, I was told that a crab has been placed in my lower abdomen so I felt like stabbing myself to remove it”.* (UD15)

## Discussion

The study sought to gain an in-depth description of dysmenorrhoea and revealed that dysmenorrhoea was an individual phenomenon. The onset and duration of pain was varied and the intensity of pain was severe. Pain was associated with a number of other signs and symptoms such as headache, diarrhoea, anorexia and nausea and vomiting. This study also aimed to explore the effects of dysmenorrhoea on participants. It was realized that dysmenorrhoea led to activity intolerance and severe pain resulted in absenteeism from work and school. It resulted in reduced work output and inattentiveness in class. The sufferers became moody, irritable, and were not able to interact effectively with other people. The pain contributed to sleeplessness and restlessness. It was revealed that severe dysmenorrhoea made some sufferers regret being females. Others wished they had early menopause, or had children. Some had misconceptions that dysmenorrhoea would lead to infertility and others had fatalistic thoughts and suicidal ideations due to the severity of the pain.

This study employed in-depth interviews with appropriate probing to gain full understanding of dysmenorrhoea characteristics and effects. The qualitative approach gave voice to students’ experience of dysmenorrhoea. The authors discussed themes to ensure that the participants’ world was accurately represented. Although the study achieved an in-depth understanding of dysmenorrhoea characteristics and effects among students, the findings like all qualitative studies, cannot be generalized. Also, samples were drawn from only two institutions in one region in Ghana and a small sample size was used to achieve saturation of data. Students from other regions may have different experiences.

The experience of menstrual pain in this study substantiates the individual nature of pain postulated by previous researchers [11]. The onset and duration of pain in this study is similar to existing literature on dysmenorrhoea [5]. However, the onset of pain after an hour of commencement of menstruation and duration of severe pain of about six hours appears to be a unique finding in this study. Associated signs and symptoms of dysmenorrhoea reported in this study are consistent with the existing literature such as nausea and vomiting, headache, diarrhoea, and backache [5]. The nausea and vomiting and diarrhoea experienced may lead to dehydration and electrolyte imbalance which can be detrimental to the health of female students with dysmenorrhoea. Therefore, individuals experiencing these problems should be cognizant of this and take steps to forestall major complications such as renal problems [14]. Although previous authors report worsened menstrual pain with a full bladder [2], some participants in this study reported pain during urination and they rather did not empty their bladder frequently.

The findings of the study indicated that dysmenorrhoea has severe negative effects on participants, similar to findings from other studies [15]. The participants were unable to perform their activities of daily living and required assistance. This finding is consistent with previous studies [5,16,17]. However, some of the University students tried to carry out their daily activities because they were independent and had to take care of their needs. The SHS students reported more debilitating effect and could not perform self-care activities. It appears that severe debilitating effect of dysmenorrhoea may improve with age leading to self-management practices [18,19]. Those with severe effects require effective pharmacologic and non-pharmacologic interventions [5]. Few Cochrane reviews confirm effective dysmenorrhoea management approaches such as use of non-steroidal anti-inflammatory drugs (NSAIDs) [20]; acupuncture [21]; and Chinese herbal medicine [22]. Thus, follow-up findings by the authors indicated students employed orthodox analgesics, herbal medicine, and non-pharmacologic approaches to manage pain. Access to analgesics and professional care were inadequate and there was inadequate knowledge on pain management [23]. The inadequate pain management contributed to the severe ill effects of dysmenorrhoea reported in this study [3,8,24].

Severe dysmenorrhoea resulted in changes in mood and social interaction among participants and these are also consistent with the existing literature [5,25]. Feelings of irritability and depression during pain were components of poor social interaction. This study emphasized that sufferers desired to be alone and sleep if possible. It is necessary for friends and family of individuals with dysmenorrhoea to understand the changes in mood, interaction, and sleep pattern so that relationships will not be marred. Individuals with dysmenorrhoea should be mindful of changes in mood and interaction and endeavour to adopt the right attitude when dealing with others. A distinctive finding in this study is the desire to be in the dark during pain with the phone and TV off. This seems to be at the extreme of social withdrawal and could lead to potential conflict if the person in pain lives with others who are very sociable.

Similar to previous studies, dysmenorrhoea resulted in absenteeism (school and work) and inattentiveness in class [1,2,12]. The finding reveals that the condition could lead to poor performance in examinations and subsequent loss of potential job or scholarship opportunities on account of poor performance. Also, since some were unable to work during dysmenorrhoea, this could result in loss of revenue as supported by previous authors [12]. In view of these ill-effects of dysmenorrhoea, some participants regretted being female and wished they were males. Others also hoped for early menopause so that they would be free from pain when their menstruation ceased. However, for those who desired early pregnancy, this could result in teenage pregnancy and may lead to complications of pregnancy. Unplanned pregnancies could lead to challenges for the family and further jeopardise the future of the pregnant teenager and the child due to possible economic hardships.

Participants associated dysmenorrhoea with infertility and this caused fear among some participants. Primary dysmenorrhoea is not commonly associated with infertility [26,27]. Rather, infertility could result from other causes some of which may result in secondary dysmenorrhoea. This study adds a socio-cultural dimension to the discourse of dysmenorrhoea. The belief that there is a “crab” in the pelvic region causing dysmenorrhoea may stem from the socio-cultural context of pain beliefs in Ghana [15]. Also, the desire to stab the body to remove the “crab” could be fatal and the desire to die due to severe pain could lead to suicidal tendencies. In view of this, individuals with dysmenorrhoea should be identified, educated and supported so that they would not have such negative thoughts.

The study highlights dysmenorrhoea as an individual phenomenon and the pain with its associated signs and symptoms varied among participants. In view of this, it is necessary for clinicians to apply the concept of individuality when handling cases of dysmenorrhoea. Health educators and clinicians should fully appreciate the effects of dysmenorrhoea, especially the psychosocial and psychosomatic effects, so that those affected can receive optimum support. Also, there should be a policy on public education on dysmenorrhea to enhance understanding of the phenomenon and its effects on women [3]. The increased knowledge could lead to effective support and management of the pain. This demands appropriate health care services that will be easily accessible to students. The ill-effects of dysmenorrhoea draw attention to the need for effective pain management and this has been reported to be inadequate within the context of the study [28,29]. Therefore, education and health policy makers should develop pragmatic policies that that would educate students on dysmenorrhoea and make pain management services easily accessible to students. Policies on effective support for students with severe dysmenorrhoea would help alleviate the ill-effect of the phenomenon on female students.

Future studies could sample students from other regions in Ghana and from rural communities to gain further understanding of dysmenorrhoea among these groups. The knowledge and attitudes of clinicians should be investigated in future studies to afford appropriate interventions to enhance dysmenorrhoea management in Ghana.

## Conclusion

Severe dysmenorrhoea has debilitating effect on sufferers and leads to absenteeism and inattentiveness in class which may result in poor academic performance. The severe pain experienced by participants in this study confirms other findings that pain management is inadequate in Ghana. Therefore, there should be policies that focus on effective pain management within the Ghanaian health system. Also, sufferers of severe dysmenorrhoea that do not respond effectively to analgesics should be supported emotionally and physically during their menstruation. The subjective nature of pain requires that students should be educated on dysmenorrhoea to enhance their self-care and coping measures. Health professionals should also be educated on the ill-effects of dysmenorrhoea and the importance of effective pain management.

## Competing interests

The authors declare that they have no competing interests.

## Authors’ contributions

LA, FD and JNCL conceptualized the study. LA collected data and LA, FD and JNCL analysed the data. LA drafted the manuscript and FD and JNCL revised the manuscript. All authors read and approved the final manuscript.
